# Effect of therapies-mediated modulation of telomere and/or telomerase on cancer cells radiosensitivity

**DOI:** 10.18632/oncotarget.26150

**Published:** 2018-10-09

**Authors:** Ganiou Assani, Yudi Xiong, Fuxiang Zhou, Yunfeng Zhou

**Affiliations:** ^1^ Department of Radiation and Medical Oncology, Zhongnan Hospital of Wuhan University, Wuhan, China; ^2^ Hubei Cancer Clinical Study Center, Zhongnan Hospital of Wuhan University, Wuhan, China; ^3^ Hubei Key Laboratory of Tumor Biology Behaviors, Zhongnan Hospital of Wuhan University, Wuhan, China

**Keywords:** cancer cells, telomere, telomerase, radiosensitivity, radiotherapy

## Abstract

Cancer is one of the leading causes of death in the world. Many strategies of cancer treatment such as radiotherapy which plays a key role in cancer treatment are developed and used nowadays. However, the side effects post-cancer radiotherapy and cancer radioresistance are two major causes of the limitation of cancer radiotherapy effectiveness in the cancer patients. Moreover, reduction of the limitation of cancer radiotherapy effectiveness by reducing the side effects post-cancer radiotherapy and cancer radioresistance is the aim of several radiotherapy-oncologic teams. Otherwise, Telomere and telomerase are two cells components which play an important role in cancer initiation, cancer progression and cancer therapy resistance such as radiotherapy resistance. For resolving the problems of the limitation of cancer radiotherapy effectiveness especially the cancer radio-resistance problems, the radio-gene-therapy strategy which is the use of gene-therapy via modulation of gene expression combined with radiotherapy was developed and used as a new strategy to treat the patients with cancer. In this review, we summarized the information concerning the implication of telomere and telomerase modulation in cancer radiosensitivity.

## INTRODUCTION

Cancer was the second leading cause of death worldwide after cardiovascular diseases [1]. In 2015, 17.5 million cancer case and 8.7 million of death were found. Cancer caused 14% of deaths in 2005 against 16% in 2015 and the number is predicted to be increased in the future. Based on the data enumerated above, cancer can be considered as an important public health which needs the strong strategies of management for its prevention and its treatment. Moreover, many kinds of strategies of treatment such as chemotherapy [2], immunotherapy [3], genetherapy [4], nanotherapy [5], radiotherapy [6], phytotherapy [7] have been developed and used to treat cancer. Among all strategies mentioned above, Radiotherapy seems more important because, in many countries, more than 50% of new cases of cancer received at least one course of radiotherapy during their lifetime [8, 9]. Although the effectiveness of radiotherapy of cancer is not negligible, radiotherapy efficacy has a limitation such as the development of radioresistance by many types of cancer [10] and occurrence of the side effects post-cancer radiotherapy [11]. Reduction of the limitation of the cancer radiotherapy efficacy via a better understanding of the mechanisms of development of radioresistance for cancer radioresistance reduction and/or decreasing of occurrence of side effects post-cancer radiotherapy is today, the main goal for many research teams of onco-radiotherapy.

Telomere and telomerase are molecular biomarkers and constitute of one of the systems implicated in sensitization of cancer to irradiation. Telomere is the prognostic and the predictive marker for stratifying patients for their post-treatment follow-up [12]. As a rational approach based on the unique role of telomerase in the cancer cell biology [13], modulation of telomere or telomerase before cancer cell irradiation reduce the limitation of cancer radiotherapy efficacy can modulates cancer radiosensitivity and the occurrence of the side effects post-cancer radiotherapy which in general, depends on many factors concerning irradiation and cancer cells or tumor tissues [14]. Based on the goal which is the reduction of the limitation of cancer radiotherapy effectiveness via especially, the understanding of how telomere and/or telomerase are implicated in radiosensitivity for radiosensitivity enhancing, we summarized many articles in which modulation of telomere or telomerase before irradiation affects cancer cells radiosensitivity which supposed to be implicated in the management of limitation of cancer radiotherapy efficacy.

## TELOMERE AND TELOMERASE IN NORMAL AND CANCER CELL

### Telomere and telomerase in the normal cell

**Figure 1:**
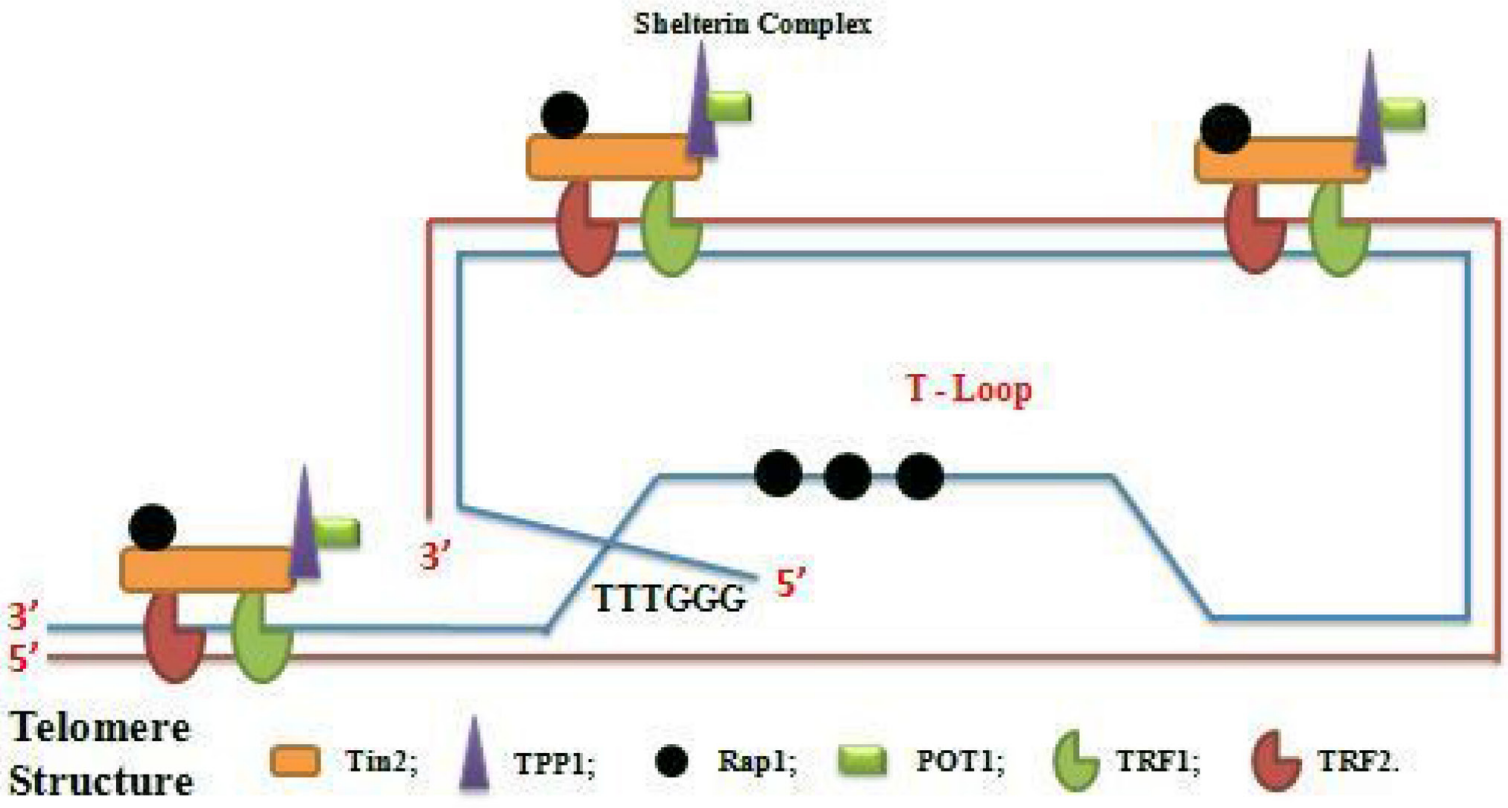
Schema of telomere structure with shelterin complex (Telosome) and T-loop formation

Telomere is the nucleoprotein structure composed of guanine-rich conserved DNA which varies in length, sequence and number of repeats. It is showed as protector of the end of the chromosome and is discovered, for the first time, in the flies and maize [15, 16]. In mammalian species, telomere is composed of TTAGGC repeat tracks that terminated in a single-stranded G-rich 3′ overhang [17–20]. The single-stranded (ss) DNA product is a few hundred nucleotides whereas the length of double-stranded (ds) telomere tracks is around 9–15Kb [21, 22]. Telomere also exists in a secondary structure called T-loop formed by the invasion of 3′ overhang into the duplex region. This is associated with protective proteins termed shelterin complex which stabilized T-loop and regulated telomere stability and homeostasis [23–26]. The shelterin complex consists of Telomere Repeat Factor1 (TRF1), Telomere Repeat Factor2 (TRF2), Repressor Activated Protein (Rap1), Protection of Telomere (POP1), Tripeptidyl-Peptidase 1(TPP1), TRF1 and TRF2 interacting Nuclear Protein2 (Tin2) which provide protection against DNA damage signals, DNA recombination or DNA end-joining processes [23]. Sheltering complex assures its function by specifically and directly binding to the telomere. TRF1 and TRF2 bind to ds DNA [27–29] whereas POT1 binds to ss-DNA [30, 31] through sequence recognition., Tin2 links to TRF1, TRF2 links to Tin2 by Protein-Protein interaction, TPP1 binds to POT1 and TRF2 to Rap1 [32, 33]. The interaction TRF1-TRF2 and POT1-TPP1 at telomere DNA are consolidated by Tin2 (Figure 1). In addition to shelterin complex, the Conserved Telomere maintenance Complex1 (CTC1), Suppressor of cdc13 1 (STN1) and telomeric pathways with STN1 (TEN1) contributed to telomere homeostasis. The telomere CTC1/STN1/TEN1 (CST) acts on telomere as a composite and functions in the replication and processing of telomere prior to affecting telomerase action. Protection effect of telomere is characterized by providing mechanism to compensate the under replication of the end of linear DNA molecule, by keeping true chromosome ends from fusing with other chromosome ends or with broken chromosome to make chimerics chromosomes, by distinguishing true chromosome ends from breaks DNA and by controlling the position of chromosome within the nucleus [34]. Moreover, the transcriptional silence of genes located close to telomere via TPE phenomenon [35], transcriptional modulation of gene at a long distance from telomere such as telomerase via TPE-OLD mechanism [36, 37], the ensuring right chromosome segregation during mitosis and definition of the number of the cell cycle, via cell cycle regulation, that a cell may undergo during its life are also the roles of telomere [28, 38–40]. In somatic cells, because of the gap between final RNA primer and end of the chromosome cannot be completed, telomere shortens after each cell division [41] with loss of 100 to 200 bases of telomere DNA per cell division [42–44]. When telomeres become critically shorten, cells undergo in a senescent state “Hayflick Limit” where cells can live for years without division [45]. Telomere length is majority regulated by telomerase activity and rarely, by Alternative Lengthening of Telomere (ALT) mechanism [46].

Telomerase is a special ribonucleoprotein enzyme which maintains telomere by neutralizing the lost of telomeric repeats at the 3′ telomeric overhang. Discovered for the first time in Tetrahymena [47], telomerase is minimally composed by a Telomerase Reverse Transcriptase(TERT), RNA Template or Telomerase RNA component (TERC) and stabilizing proteins which including dyskerin (DCK1) and TCHB1 [23, 48]. Telomerase activity maintains telomere length but not totally prevents telomere attrition [49–52]. The mechanism of action of telomerase can be divided into three steps. In the first step, the 3’matrix end of the chromosome of the short telomere binds to RNA domain, in the second step, occurrence of elongation which is a direct addition of nucleotide and last step, translocation which enables repeated use of the same binding site [53–61]. The human Telomerase Reverse Transcriptase (hTERT) catalytic subunit is a polypeptide which consists of 1132 amino acids and plays an important role in cell lifespan. At least, three domains can be distinguished in the structure of TERT structure. RNA-binding domain of telomerase knows as TRBA, reverse transcriptase domain and the poorly conserved C-terminal domain [62–64]. Certain TERT contain an additional N-terminal which facilitate the addition of telomere repeat by its implication in the process of primer binding [62, 65]. Telomerase also contains the region which acts as a template for telomere synthesis [66, 67]. Telomere RNA (TER) structure contains certain conserve elements such as template region, the pseudoknot, the trans-activating domain and the domains required to ensure *in-vivo* stability, meaning that TER contains the essential elements for telomerase activity, assembly, localization and stability of RNA. Apart from the telomerase activity of telomerase, telomerase and telomerase component have an alternative functions in cell life such as telomerase nuclease activity because the length of the final products depends on the template region of telomerase RNA [68], transferase activity via stimulation of certain small molecules [69, 70], mitochondrial function activity via implication of hTERT in replication and repair of mtDNA [71], DNA damage activity [72] and regulation of gene activity [73–75]. According to the relationship between telomerase and telomere and their roles in cell function and life, dysfunction of telomere and /or telomerase can lead to dysfunction of a cell (disease).

### Telomere and telomerase in human diseases especially cancer

Telomere and telomerase, via their dysfunction, are implicated in several human diseases such as chronic lung disease [76–78], chronic obstructive pulmonary disease and idiopathic pulmonary fibrosis [79–81], diabetes [82, 83], autoimmune disease (rheumatoid arthritis, systemic Lupus erythematosus, sclerosis) [84–88], renal failure (chronic kidney disease) [89], cardiovascular disease [90–92], Parkinson disease [93, 94], chronic infection [95, 96], obesity [97], cancer [28], etc.

As mentioned above, telomere become short after each cell division because of «end replication problem» and this telomere shortening is a natural phenomenon in cell viability and chromosome stability where the lagging strand DNA synthesis cannot be completed all the way to the very end. In this case, increasing the division of the cells leads to very short telomere which causes the DNA damage responses that trigger cellular senescence [98]. The cells in senescence phase have short telomere and are characterized by inhibition of cell proliferation. The lost of telomere quality, in that case, promotes the DNA repair system and tumor suppressor protein _P_53 which stimulates _P_Rb. Activation of _P_ and _PRb_ leads essentially to irreversible growth arrest. However, the cells which gain additional oncogenic changes such as _P_53 loss can pass senescence step and can continue to divide. This initiates the crisis step associated to chromosome end-to-end fusion (new dysfunctional step) and increasing of the cell death [99]. However, very few human cells (1 in 10^5^ to 10^7^) can continue the division and in this case, with an acquisition of cell immortality or cancer initiation ability [100]. At this step, certain cells have very short telomere without a genomic instability which is maintained by reactivating and increasing of telomerase expression or in the rare case, by activating the telomerase-independent mechanism (ALT) [101]. Several studies demonstrated the correlation between telomere shortening and cancer risk with cancer type -dependent [102, 103]. In the population level, it has been reported that patients with short telomere in peripheral blood cell have a high risk to develop cancer [104]. However, the shortening of telomere is supposed to protect against the malignant transformation of the cells by limiting cell proliferation. For its confirmation, It’s found that peoples with short telomere length have a low risk of melanoma development than control [105] suggesting that chromosome instability is indispensable in the occurrence of cancer initiation mediated by telomere dysfunction and only telomere shortening induced chromosome instability is implicated in cancer initiation and progression [106–109]. This confirms the discover mentioning that telomerase is reactivated and overexpressed with bypass crisis step where chromosome end fusion, rearrangement of the chromosome, malignant transformation have occurred. *Kim NW et al.* reported that high levels of telomerase expression associated with telomere shortening is detected in most of human cancer to assume telomere elongation and maintenance whereas it is absent in most of the normal somatic cells or tissues [28]. Although telomerase has a high preference for short telomere [110, 111] which is implicated in cancer initiation, its expression is controversial because some cancer cell does not have a high level of telomerase expression which may be due to ALT mechanism. Because telomerase consists of catalytic subunit telomerase reverse transcriptase (TERT), Telomerase RNA component (TERC) and telomerase complex associated protein, upregulation of telomerase expression is correlated with increasing of copy number of hTERT which is strongly positive in tumor cell [112] and correlated with telomerase activity, cancer initiation and progression [113–115]. Based on the important role of telomere shortening induced genomic instability and telomerase in cancer development, they can be considered as a good target for reinforcement of strategies for improvement of cancer therapy especially cancer radiotherapy.

## THERAPIES-ASSOCIATED WITH TELOMERE OR TELOMERASE MODULATION AND CANCER CELLS RADIOSENSITIVITY

### Telomere and telomerase as good targets for enhancement of cancer radiosensitivity

Several studies reported the link between short telomere and radiosensitivity [116, 117]. In human, irradiation induces damage in people with short telomere than people with long telomere [118, 119]. In cell level, *Zhong YH. et al.* reported that there is a negative correlation between radiosensitivity of 15 human carcinoma cell lines from different tissues and their telomere length [120]. In the same way, *Cabuy E. et al.* showed that high radiosensitivity human cells have short telomere than the normal cell [121]. *McIlrath J. et al.*, also reported that murine lymphoma cells L5178Y-S which have 7 Kb is more radiosensitive than L5178Y-S having 48 Kb [116]. The negative correlation between radiosensitivity and telomere shortening may be caused by telomere shortening associated to chromosome aberration [122, 125] and chromosome aberration is associated to radiosensitivity [124], by chromatin structure change where the access of ATM to its target chromatin is limited [117, 123, 124]. Moreover, it has been reported that late generation of mTR(−/−) such as G5mTR(−/−) mice and Terc−/− mice exposed to γ-Ray shown high mortality via increase rate of apoptosis and cytogenic damage [117, 124], suggesting that there is also a negative correlation between telomerase and cancer cells radiosensitivity. Based on those data, telomere and telomerase can be considered as good biomarkers for cancer radiosensitivity and their modulation can enhance cancer response to irradiation.

### Telomere dysfunction and radiosensitivity

Several telomere or telomerase modulation approach such as Telomere Homolog Oligonucleotide, G-quadruplex Ligand, targeting of telosome or another Telomere maintenance proteins has been demonstrated to be implicated in telomere dysfunction -associated with modulation of cancer radiosensitivity.

### T-Oligos and G4-Ligand

Telomere Homolog Oligonucleotide called T-Oligos and G-quadruplex Ligand or G4-Ligand are developed and used to induce telomere dysfunction mediated enhancement of cancer radiosensitivity. Telomere Homolog Oligonucleotide called T-Oligos mechanism of action is to accumulate in the nucleus and rapidly promoted DDRs at telomere-mediated by _P_53, ATM, E2F1, cdk2 and _P_95/NBS1 which finally leads to cell cycle arrest, senescence, apoptosis [126–130]. Because of its specific anti-cancer effect, T-Oligos is showed to highly affected viability and growth of cancer lung cell, melanoma, prostate, ovarian, breast and colorectal cancer [129, 131–134]. T11 is one of T-oligos which consists of 11 oligonucleotides and has an anti-cancer effect in several types of cancer [135–136] *in-vitro* and *in-vivo* [137, 138]. It enhances the anti-cancer effect of irradiation [139]. G-quadruplex or G4 is a structure which forms naturally in telomere region by folding of non-coding repeat sequence of guanine-rich DNA (Telomeric ends called G-rich ends of the chromosome). By its ligand, G4 stabilization can prevent telomere elongation which leads to telomere embrittlement [140]. G4-Ligand is G4 stabilizing ligand used as a potential treatment for cancer development and progression [141]. Several G4 ligands are developed and used as cancer therapy whereas very few are used in combination with radiotherapy. It is reported that G4-ligand binds to G4 DNA and highly sensitizes cancer cell to irradiation [142]. Pt-ctpy also is kind of G4-ligand with a good affinity for G4 DNA [143] and belongs to tolyterpyridine metal [144, 145]. *Merle P et al.* reported that Pt-Ctpy induced reduction of GBM and NSCLC cell proliferation in concentration-dependent-manner, induced the accumulation of S-phase cells, G2/M phase cell and apoptosis cells [142]. Pt-Ctpy is well tolerated without toxic effect where it’s used alone and it increased irradiation effect *in-vitro* and *in-vivo* when it is used in combination and before radiotherapy. TAC is another G4 Ligand and contains 70% of TAC-Me2 and 30% of TAC-Me3. It is reported as inhibitor of cancer cell (GBM) proliferation in a dose-dependent manner via a minimal effect on cell cycle and apoptosis after one week alone treatment. However, the therapeutic dose (5 Gy) of TAC which does not have an effect on cell cycle and apoptosis induced high sensitization of GBM cell to irradiation [146]. The pentacyclic acridine RHPS4 is also G4 Ligand implicated in telomere no protection (dysfunction) and blockage of cell proliferation [147–149]. By using the comparison between the survival curves, *Berardinelli F. et al.* reported that RHPS4 enhanced cancer cell radiosensitivity. In the same report, U251MG radioresistant cancer cells pretreated 120 h with 0.2 µM of RHPS4 before 2 Gy irradiation showed 53% of reduction of survival whereas unpretreated cell showed 20% of decreasing and RHPS4 combine with irradiation (0.2 µM of RHPS4 +3 Gy) activate a transient G2 phase blockage which leads to the cell proliferation reduction at 0.61 proportion and 0.84 proportion of cell proliferation reduction for (3 Gy) [150].

### Telosome modulation and cancer radiosensitivity

As telomere maintenance element, telosome (telomere sheltering complex protein) is a complex of 6 proteins (TRF1, TRF2, RAP1, TIN2, TPP1 and POT1) which modulation is one of the ways to promote telomere dysfunction associated to cancer high radiosensitivity.

*Zhou YF et al*. reported that POT1 which is one of telosome is implicated in the regulation of cell radiosensitivity [151]. Otherwise, the POT1 expression is in positive correlation with telomere length because patients with high level of POT1 have long telomere and were photon irradiation resistant [152]. Downregulation of POT1 by using siRNA increase human cancer cell radiosensitivity. TRF2 also is one of telosome and is implicated in the modulation of cancer radiosensitivity. It is reported that inhibition of TRF2 expression enhanced the effect of 2.5 Gy of γ-Ray irradiation by decreasing scurvies cell fraction [153] and by increasing γ-H2AX foci leading to the reduction of telomere protection from irradiation consequence of high radiosensitivity [154]. TPP1 is one of the radioresistance proteins because it is overexpressed in the radioresistant cancer cells and its ectopic expression confers radioresistance ability to cancer cell [155]. *Zhou YF et al.* reported that modulation of TPP1 modulates telomere homeostasis and radioresistance of human colorectal cancer [156]. It’s also reported that its suppression enhanced cancer radiosensitivity in telomerase negative cell by inducing telomere dysfunction [157]. However, the role of Rap1, TRF1 and Tin2 which are also a part of shelterin complex, is not known yet in cancer radiosensitivity. Then, it will be good to encourage more research concerning that to clearly master the role of those proteins particularly and generally, telosome in cancer response to irradiation in the relationship with telomere function.

### Other telomere maintenance proteins and cancer radiosensitivity

The telomere maintenance component 1 (CTC1) is the third member of the CST (CTC1-STN1-TEN1) complex binding to telomere and assume its integrity [158]. CTC1 knock-down promotes great telomere loss [159, 160] and it implicated in the modulation of radiosensitivity. *Zhou YH et al.* demonstrated that CTC1 expression is inhibited in radiosensitive human melanoma cells compared to radioresistant cells and its total inhibition increased cell radiosensitivity by promoting telomere shortening and apoptosis [161]. Ku80 is one of the subunits of the Ku80/Ku70 heterodimer which is implicated in telomere maintenance by binding to the DSB ends and by initiating its repair via DNA-PKCs recruitment [162, 163]. It’s reported that its deficiency leads to cancer cells sensitization to irradiation and Ku80 mutation also leads to telomere ends repair prevention [164] which decreased telomere length and enhanced the response of many cancer cell line to irradiation [165]. Otherwise, *Zhou FX et al.* reported that down-regulation of Ku80 by using siRNA enhanced the radiosensitivity of telomerase deficiency cell U2OS by inducing telomere shortening [166]. The high mobility group box1 (HMGB1) is a ubiquitous chromatin-associated protein which implicated in non-homologous end-joining, mishmash repair [167] and telomere maintenance. It’s implicated in cancer progression and its Knock-down in mouse embryonic fibroblast (MEFs) leads to telomere dysfunction [168]. Moreover, *Zhou YF et al.* reported that inhibition of HMGB1 enhanced human cell radiosensitivity via inhibition of repair kinetics of DNA damage induced by irradiation, increasing of apoptosis, decreasing of the proportion of cell in S-phase and induction of telomere shortening [169]. WRAP53 protein is implicated in telomere elongation caused by telomerase and is highly expressed in a cancer cell [170]. Depletion of WRAP53 reduced telomere length without affect telomerase activity [171–173] suggesting that it has a high relationship with telomere maintenance. WRAP53 modulation can modulate cancer radiosensitivity because of the negative correlation which exists between WRAP53 and cancer cells radiosensitivity. Decreasing of WRAP53 is associated to high radiosensitivity. *Xie CH et al.*, reported that in Hep2 cell, transfection of a cell by phWRAP53-siRNA for inhibition of WRAP53 expression before irradiation showed high radiosensitivity via telomere shortening than non-transfected cells [174]. All data mentioned above indicate that telomere play an important role in cells function and its dysfunction is lethal for cancer cells which can lead to enhancement of cancer therapy such as cancer radiotherapy. Basing on the relationship between telomere and telomerase, targeting telomerase may also be one way to promote telomere dysfunction-induced radiosensitivity.

## TELOMERASE MODULATION AND CANCER CELLS RADIOSENSITIVITY

Several strategies or techniques such as oligonucleotide inhibitor, the small-molecule telomerase inhibitor, Immunotherapy approach (Vaccinotherapy), Telomerase direct or indirect gene therapy (RNA interference) and phytotherapy are developed and used in cancer therapy. Among them, few are used, as telomerase inhibitor by targeting of telomerase RNA (TR) or telomerase reverse transcriptase (TERT), in combination with irradiation to enhance cancer cells response to irradiation via telomere shortening (Telomere dysfunction).

### Telomerase activity Oligonucleotide inhibitor and cancer cells radiosensitivity

Imetelestat (GRN163L) is a 13-mer oligonucleotide which reported to suppress catalytic activity of telomere by targeting the template region of hTR [175, 176]. Its anti-cancer effect is reported on breast cancer [177], prostate cancer [178], glioblastoma [179], myeloma leukemia [180] and it is also showed as cancer radiosensitivity modulator. *Zhigang G et al.* reported that imetelestat treatment before irradiation enhanced esophageal squamous cancer cells response to irradiation by inducing DNA break (apoptosis) and reducing cell proliferation *in-vitro* and *in-vivo* [181]. Similar results are discovered by *Gomez-Millan J et al.* on breast cancer cell where treatment of MDA-MB-231 cancer cell with GRN163L enhanced cancer radiosensitivity *in-vitro* and *in-vivo* [182] via telomerase activity and telomere length inhibition. *Sylvain T et al.* also showed that imetelestat increased the response of a tumor to irradiation illustrated by tumor volume reduction and inhibition of telomerase activity [183]. ASODN is another oligonucleotide inhibitor which inhibits telomerase activity via targets of human telomerase RNA and called hTR ASODN. *Zhou YF et al.* reported that hTR ASODN increased esophageal squamous cancer cells sensitivity to irradiation by down-regulating telomerase activity and increased human neuroglioma cell (U251) sensitivity to irradiation by inducing DNA damage and by reducing cell proliferation [184]. In 2005, *Zhonghua Yu et al.* reported the similar results on nasopharyngeal carcinoma cells where cells treated with the combination of hTR ASODN and irradiation showed high sensitivity to irradiation via reduction of their proliferation and their telomere length [185].

### Direct or indirect gene therapy targeting telomerase and cancer radiotherapy

Direct gene therapy of telomerase means the use of RNA interference (siRNA, shRNA, miRN…; etc) to post-transcriptionally silence telomerase gene expression which leads to telomerase mRNA reduction. This kind of telomerase reduction can lead to telomere shortening and can be implicated in radiosensitivity modulation because certain TERT or TERT promoters’ mutations are associated to telomere length and predict poor survival and radioresistance [186]. Mice deficient in the RNA component of Telomerase is high radiosensitive [117, 124] and HCT116 cells with hTERT allele disruption (hTERT +/−) called haplo insufficient had a reduction of telomerase activity, telomere length and are more radiosensitive [187]. The similar results were reported by several discovers where direct inhibition of telomerase expression by transfecting cancer cell with RNAi and with shRNA before irradiation enhanced the effect of irradiation via reduction of telomerase activity and/or telomere length [188–192].

Many researches are carried out concerning the indirect target of telomerase which is the target of a protein implicated in telomerase function. Survivin is a member of the inhibitor of apoptosis (IAP) protein family and is highly expressed in most of the cancer cells whereas it is undetectable in normal cells [193, 194]. *Zhang HZ et al*. reported that the use of siRNA to inhibit telomerase activity in control of surviving promoter enhanced radiotherapy effect in Hela cell *in-vitro* and *in-vivo* [195]. Ubiquitin-Conjugating Enzyme called UBE_2_D_3_ or E_2_D_3_ is a key component in ubiquitin-proteasome system and is reported to lowly express in many cancer cell line [196]. *Zhou YF et al.* demonstrated that UBE2D3 is negatively correlated to radioresistance [197–200] and its inhibition decreased radiosensitivity by increasing hTERT expression and telomerase activity in MCF-7 cell [199]. The author also reported that UBE2D3 overexpression enhances radiosensitivity by reducing telomerase activity and telomere length in esophageal cancer cell *in-vitro* and *in-vivo* [200]. Tankyrase 1(TNKS1) is a protein required for telomerase activity [201–203]. Indirect telomerase inhibition by using siRNA TNKS1 against TNKS1 leads to telomere uncapping and increasing of cancer cell ionizing irradiation sensitivity [204]. The latent membrane protein (LMP1) encodes by Epstein-Bar Virus (EBV) is suggested to be one of the major oncogenic factors in nasopharyngeal cancer cell line and is implicated in hTERT activation. EBV-LMP1 DNAzyme (D_2_L) is a DNA enzyme which binding to their target RNA via Wastor-Crick base-pairing and cleaves the mRNA of LMP_1_ [205]. It is reported that activation of hTERT is mediated by LMP1 and targeting LMP1 by using D2L leads to increase of radiosensitivity via inhibition of hTERT expression and telomerase activity [206].

### Phytotherapy associated with telomerase targeting and cancer radiosensitivity

Phytotherapy is the use of the vegetable drug or vegetable extraction to treat diseases including cancer [207]. Many of them showed an anti-cancer effect by targeting telomerase expression or telomerase activity while few of them are used in combination with radiotherapy for radiotherapy effect enhancement. Panax ginseng is one of the most common herbals in medicine and ginseng saponins (ginsenosides) are its major active components. As an anti-cancer product, ginseng saponing is reported to have an anti-tumor effect by downregulating telomerase activity [208, 209]. It is also reported that panax ginseng (ginseng sponing) modulated radiosensitivity of cancer cell. *You JS. et al.* reported that the use of ginseng in combination with radiotherapy more reduced the survival rate of tumor cell (66.7%) vs (75.3%) and (76.1%) for ginseng and irradiation alone treatment respectively. Suggesting that the synergic use of ginseng and radiotherapy may increase cancer cells radiosensitivity and may decrease irradiation side effects [210]. Resveratrol is a major component of polyphenol from grapes and it was used for many disease treatments including cancer [211–215]. It is also reported that its relative high concentration substantially inhibited cancer proliferation and telomerase activity in human colorectal cancer and in breast cancer cell line [214, 216]. Resveratrol enhanced EOL1 cancer cell radiosensitivity by inducing apoptosis [217], NSCLC cancer cell radiosensitivity by increasing ROS generation and DNA DSBs [218] and MCF-7 cancer cell radiosensitivity by inducing cell cytotoxicity and activating of different pathways of cellular death [219]. Curcuma is polyphenol contains in Curcuma Longa and its anti-proliferative effect was reported in many types of cancer cell line such as head and neck squamous cancer cell, breast cancer, prostate cancer, lung cancer, pancreas cancer [220–226]. Anticancer effect of Curcuma is associated with apoptosis induction and reduction of telomerase expression or activity in several cancer cell lines [227–230]. It is reported to enhance cancer radiosensitivity [217] via the different mechanisms such as suppression of NF-kb activity [231]. Epigallocatechin-3-gallate (EGCG) is a major polyphenol (more than 50% of total polyphenol) contains in green tea [232]. As a possessor of specific anti-cancer ability [233, 234], EGCG showed its anti-proliferative effect via the different mechanisms like induction of apoptosis, inhibition of cell migration, inhibition of telomerase expression or telomerase activity and telomere length [235–240]. EGCG used in combination with X-irradiation resulted in enhancement of cancerous cell line response to irradiation [217, 241].

However, the mechanisms of synergic effect of phytotherapy products mentioned above (ginseng, Curcuma, resveratrol, EGCG) combined with irradiation treatment does not include telomere or telomerase targeting. It will be good to encourage more research concerning that to know if targeting telomere or telomerase is included in the mechanism of their synergic effect on cancer cell radiosensitivity.

## ADVANTAGES OF THE USE OF THE COMBINATION OF THERAPIES TARGETING TELOMERE OR TELOMERASE AND CANCER RADIOTHERAPY

**Figure 2:**
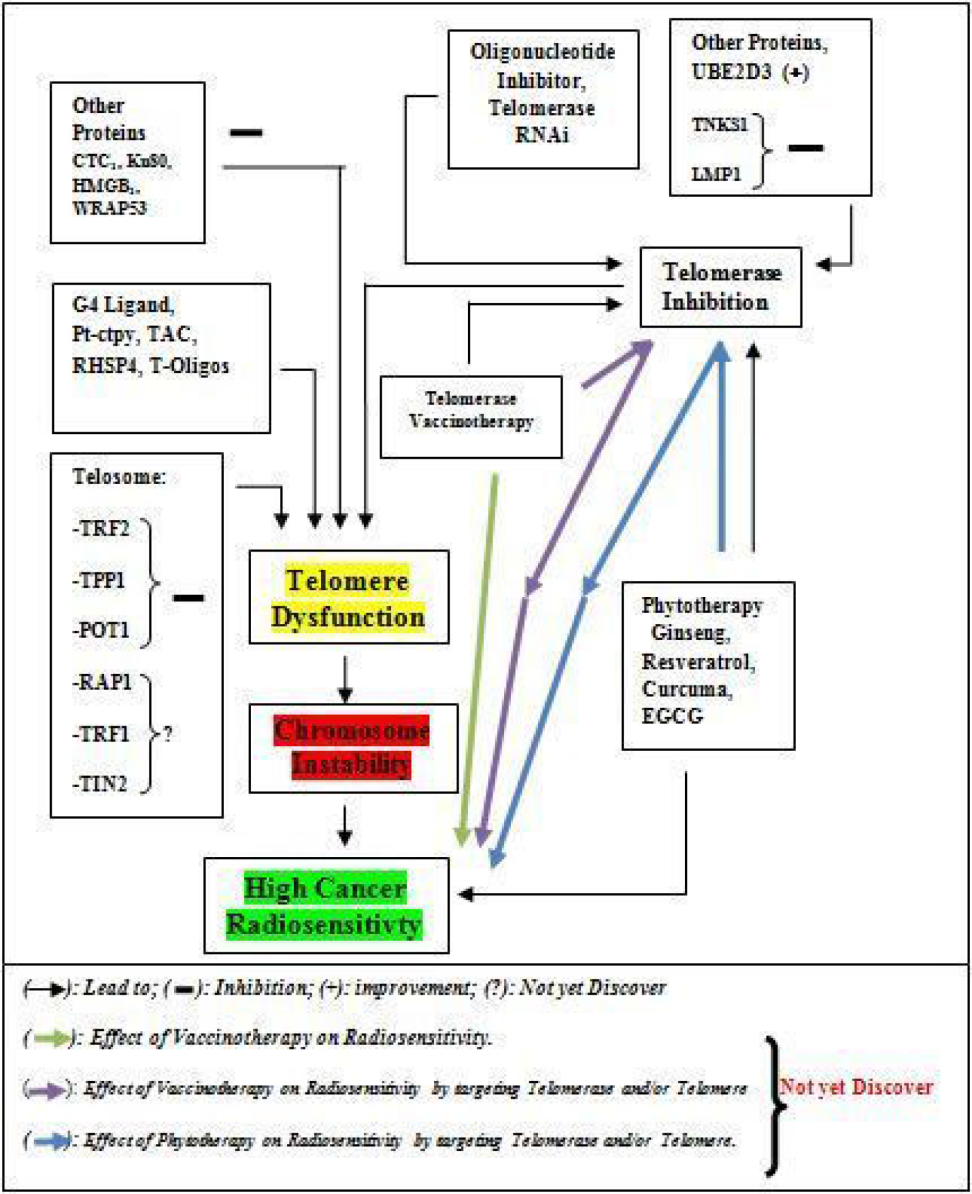
Schematic diagram of link between therapies associated with telomere or telomerase modulation and Cancer Radiosensitivity

Radiotherapy for curative treatment has an important part in cancer treatment with many advantages and disadvantages. The main disadvantages of radiotherapy are the damage of normal tissues or cells which depend on the volume of tissue, the total dose of radiation, the dose per fraction of radiation, irradiation delivery method, factor and comorbidities of the patients [14, 242]. The damage of normal tissues by radiotherapy can lead to cardiovascular diseases, a pulmonary complication, infertility, endocrinopathy abnormality and second malignancy [243, 244], dermatitis [245], and gastrointestinal diseases [246], which are known as side effects of the post-cancer radiotherapy, are a major health and socio-economic problems. Combination of other therapy which modulates telomere and/or telomerase associated with a reduction of cancer proliferation and radiotherapy (Figure 2) is one of the strategies for reducing total dose of irradiation which may reduce side of effect induce by high total irradiation or per fraction irradiation dose. Based on these advantages, it will be good to complete the list of targeting telomere or telomerase mediated high radiosensitivity treatment such as vaccine-therapy (immunotherapy) which can be used in combination with irradiation treatment to improve irradiation effect. In our case, telomerase vaccine-therapy leads to high expression of telomerase in cancer treatment *in-vitro* and *in-vivo* manner and as a tumor neo-antigen to stimulate the cancer cells deaths since it is reported that TERT is a tumor-associated antigen (TAA) which caused antitumor CD8+ cytotoxic lymphocyte (CTL) response in several types of tumor [247]. Several telomerase vaccines are developed and used in the treatment of cancer in fundamental and clinical research [248] where they showed a high anti-tumor effect by activating an immune system against telomerase for its destruction [249]. For this, more research should be encouraged to explore more on the combination of telomerase vaccine and irradiation in future and application of that kind of combination for cancer treatment could enhance radiotherapy effect and reduce side of effect post-cancer radiotherapy.

## CONCLUSIONS

Radiotherapy is one among the of cancer treatment and plays an important role in treating cancer. As other cancer treatments, radiotherapy efficacy has also certain limitation such as cancer radiotherapy resistance and side of effect post-cancer radiotherapy. Telomere and telomerase are cancer markers and are implicated in cancer development, cancer treatment and limitation of cancer treatment effectiveness such as cancer radiotherapy limitation especially cancer radio-resistance. Combination of two treatments by modulating telomere or telomerase before radiotherapy treatment improves cancer radiosensitivity if only the modulation of telomere or telomerase is associated with chromosome instability. Meaning that chromosome instability is the key factor of telomere or telomerase modulation mediated high cancer radiosensitivity. However, several researches need to be done concerning the reinforcement of cancer radio-gene-therapy used before and certain kind of cancer treatment such as vaccine-therapy and phytotherapy need to be used in combination with radiotherapy to expand the group of therapies mediated telomere or telomerase targeting (chromosome instability) which could be used in combination with radiotherapy for reducing its effectiveness limitation especially, via the enhancement of cancer radiosensitivity.
